# Simulation of Dynamic and Meta-Dynamic Recrystallization Behavior of Forged Alloy 718 Parts Using a Multi-Class Grain Size Model

**DOI:** 10.3390/ma14010111

**Published:** 2020-12-29

**Authors:** Christian Gruber, Peter Raninger, Aleksandar Stanojevic, Flora Godor, Markus Rath, Ernst Kozeschnik, Martin Stockinger

**Affiliations:** 1 Materials Center Leoben Forschung GmbH, 8700 Leoben, Austria; peter.raninger@mcl.at; 2 Voestalpine BÖHLER Aerospace GmbH & Co KG, 8605 Kapfenberg, Austria; Aleksandar.Stanojevic@voestalpine.com (A.S.); flora.godor@voestalpine.com (F.G.); 3 TU Wien, Institute of Materials Science and Technology, 1060 Vienna, Austria; markus.rath@stud.tuwien.ac.at (M.R.); ernst.kozeschnik@tuwien.ac.at (E.K.); 4 Department for Product Engineering, Montanuniversität Leoben, 8700 Leoben, Austria; martin.stockinger@unileoben.ac.at

**Keywords:** alloy 718, microstructure modeling, multi-class grain size, dynamic recrystallization, meta-dynamic recrystallization, screw press, hammer forging

## Abstract

Dynamic and meta-dynamic recrystallization occur during forging of alloy 718 aircraft parts and thus change the microstructure during a multistep production route. Since the prediction of the resulting grain structure in a single grain fraction is not able to describe microstructures with bimodal or even multimodal distributions, a multi-class grain size model has been deployed to describe the recrystallization mechanisms during thermomechanical treatments and predict the resulting grain size distributions more accurately. As forging parameters, such as temperature, strain rate and maximum strain influence the flow curve and consequently the recrystallization behavior, a series of double cone compression experiments has been carried out and used to verify and adapt the material parameters for the multi-class grain size model. The recrystallized fractions of the numerical and experimental results are compared and differentiated in view of the recrystallization mechanism, i.e., dynamic and meta-dynamic recrystallization. The strong dependence of the recrystallization kinetics on the initial grain size is highlighted, as well as the influence of different strain rates, which shall represent typical forging equipment.

## 1. Introduction

During the production of aircraft parts from alloy 718, the material undergoes several forging steps and heat treatments [1,2]. In case of the forming equipment, such as screw presses and hydraulic or counterblow hammers, the difference in the energy input leads to difference in the recrystallization behavior of the material and thus leads to a variation in the microstructure of the final component [3,4,5,6,7,8,9,10,11,12,13,14]. An optimization of the process parameters to adjust the microstructural and mechanical properties is essential. For this reason, a digital model of the complete thermo-mechanical process sequence has been designed and experimentally validated to replicate the evolution of the microstructure [15,16,17]. The initial grain size distribution at the beginning of the forging process and forging parameters with respect to the temperature, strain rate and maximum strain are systematically varied in order to study the general recrystallization behavior [18,19,20,21]. The focus is set to the separation of dynamic (DRX) and meta-dynamic (MDRX) recrystallization combined with their kinetics [3,22,23,24,25,26,27]. The variation in the microstructure is based on simulations with industrially measured process data and is applied in a multi-class grain size model [28]. For this purpose, a comprehensive literature review was carried out to adapt flow curves at different strain rates and initial grain sizes. This study enables specific parametrization of the semi-empirical modeling approach. The resulting multi-class grain size model with specific thresholds in terms of the strain where recrystallization sets in for different forming conditions is used to compare the recrystallized fractions of a test series of double cone compression tests. An adaptation to the extreme values using further literature sources on this topic is also achieved by a cutoff limit of the strain threshold to the maximum yield point from the flow curves [3,21,26].

To validate the model, the samples (double cones) from the compression tests were evaluated by electron backscatter diffraction (EBSD) analyses with respect to recrystallization and grain size distribution [29]. These findings are compared to the results of the multi-class grain size model calibrated to the threshold values from ASTM standard E112-96 [30]. The knowledge of the recrystallization behavior due to different forging processes is used to reduce the development time of aircraft parts and to set up the different forging equipment to work under optimal process conditions for optimal component properties right from the first produced part.

## 2. Materials and Methods

To determine the DRX and MDRX behavior of alloy 718, a number of double cone compression tests were performed on a Servotest TMTS (Thermo-Mechanical Treatment Simulator) (Servotest, Egham, UK) [31]. Special attention was given to a variation of temperature, strain rate and holding times after deformation. To determine the influence of the grain size, two different initial microstructures were chosen. The simulative reproduction of the test series was carried out with the commercial finite element method (FEM) software package DEFORM v12.0 (Scientific Forming Technologies Corporation, Columbus, OH, US). The microstructural evolution was calculated with the multi-class grain size model [28] based on the local evolutions of temperature and strain.

## 2.1. Alloy 718

The high-strength nickel-based super alloy 718 was used as material to describe the recrystallization behavior and for demonstration of the multi-class grain size model. The material state for sample manufacturing and testing was a wrought triple melt bar material with a diameter of 254 mm, which was homogenization annealed, upset and long-forged. The typical chemical composition of alloy 718 is shown in Table 1. Depending on the process route during the various production steps, more or less pronounced variations of the microstructure occur within the bar material. This concerns grain sizes, grain size distributions and precipitations in terms of shape, size and chemical composition. Thus, the position within the bar affects the recrystallization behavior and a differentiation into two extrema was chosen. Further information will be given in Section 2.2.3.

## 2.2. Double Cone Compression Test

Double cone compression tests were performed for defined strains and strain rates. The advantage of this test method is the linear increase of the effective strain over the radius at a constant local strain rate. In order to simulate the real process parameters of screw presses [3,8,11] and forging hammers [5,12,13,14], the following temperatures, strain rates and two differentiated initial structures were selected and summarized in Table 2. In order to differentiate between DRX and MDRX [3,9,21,23,24,25,26], the same forming conditions were applied to all tests where quenching was conducted either directly after forming or after 5 and 15 s holding time. This allows the exact determination of the fraction and the kinetics of the MDRX behavior of the respective samples under given conditions.

## 2.2.1. Double Cone Geometry

**Figure 1:**
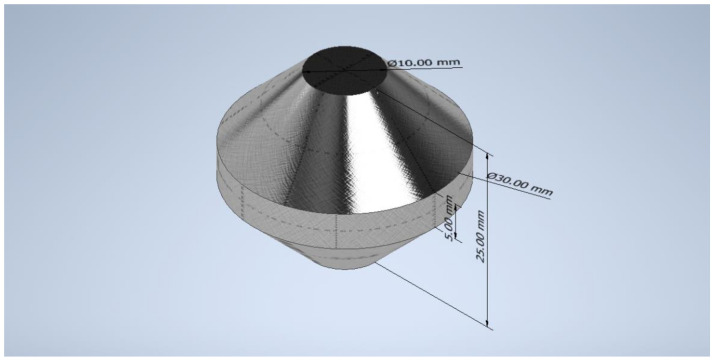
Double cone geometry used for compression tests.

Double cones were selected as sample geometry due to the advantages of linear increasing strain during forming as well as the gradient of decreasing strains over the radius of the cone. Furthermore, the local strain rates over the globally introduced deformation with constant values during the deformation is a further facilitation for the tracking of defined points with a defined strain of interest. Consequently, one sample provides a multitude of different local effective strains, which can be evaluated separately with a single compression test. For the geometry of the double cone, a height of 25.00 mm and a maximum diameter of 30.00 mm was chosen, whereas the head diameter is 10.00 mm. The sample geometry is shown in Figure 1.

## 2.2.2. Servotest TMTS

**Figure 2:**
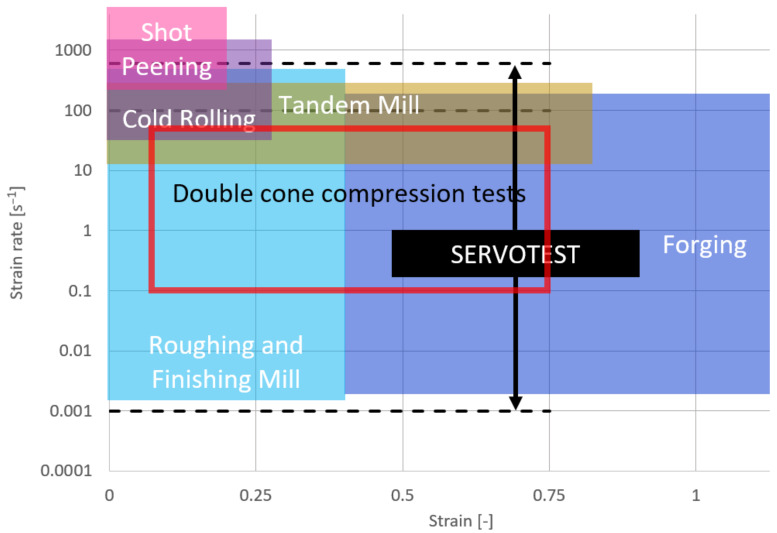
Typical process parameters in metal forming.

The series of compression tests was performed on a Servotest TMTS [31]. The integrated furnace system allows an isothermal temperature control during the process. The adiabatic heat through deformation was recorded with a thermocouple in the double cone specimen and compared to the results of the simulation. All compression tests were performed until an end height of the specimen of 17.00 mm, which corresponds to a compression of 8.00 mm and, predicted from simulation, local maximum strain of 0.74 in the core of the sample. Comparing the chosen strain rates with the characteristics of different forming processes, as shown in Figure 2, wherein the red rectangle marks the area of testing conditions, a broad range of different forging equipment can be experimentally simulated.

## 2.2.3. Initial Grain Size Distribution

In order to determine the influence of the initial grain size on the recrystallization behavior, two different initial grain sizes and distributions were selected. This variation in the initial grain structure influences the flow stress during the forging process of the material and is also important for the local dislocation density development which strongly influences metallurgical processes such as recrystallization.

**Figure 3:**
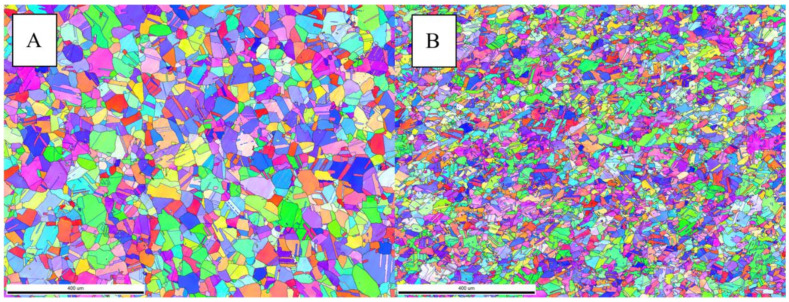
Electron backscatter diffraction (EBSD) inverse pole figure (IPF) maps of microstructures (**A**,**B**).

**Figure 4:**
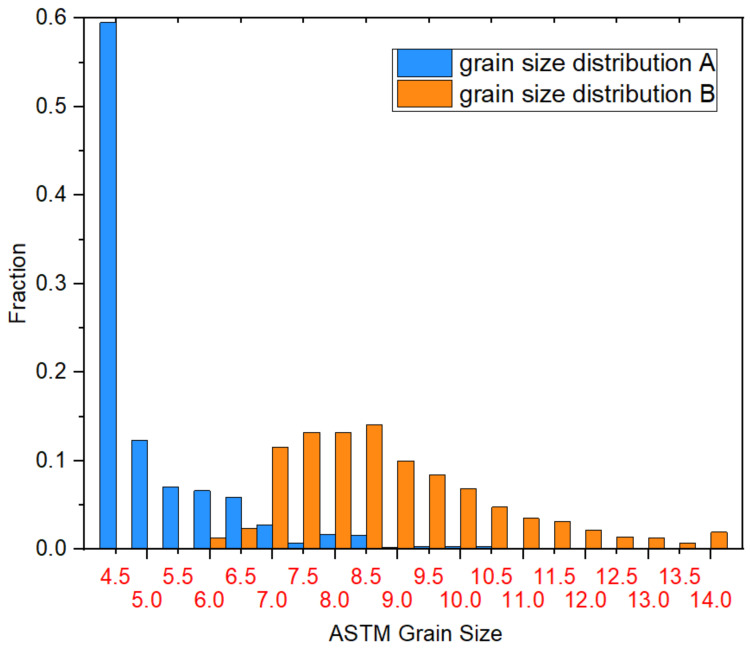
ASTM grain size distributions of microstructures A and B.

An overview of the two different microstructures is given in Figure 3 with two EBSD inverse pole figure (IPF) grain maps. The difference in the grain size distributions is visible and expressed in numbers in Figure 4. The values with removed twins are used as starting point to define the recrystallized fractions after the compression tests.

## 2.3. Simulation and Multi-Class Grain Size Model

**Figure 5:**
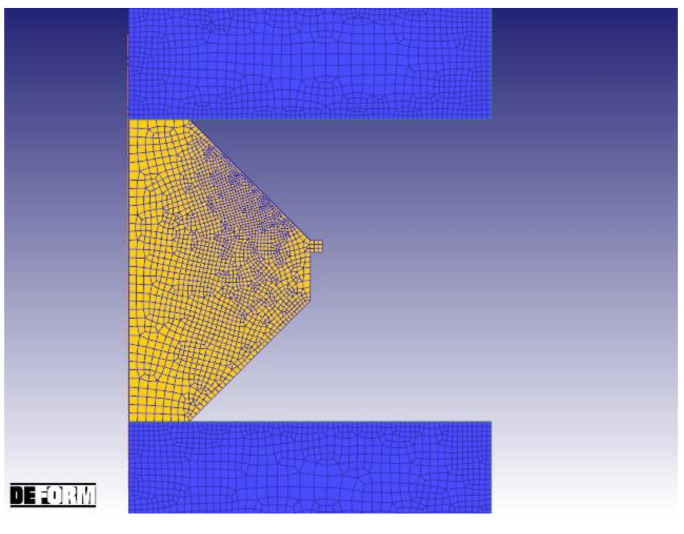
Double Cone simulation setup.

FEM simulations were set up in the software DEFORM for the before mentioned double cone compression tests. Therefore, all process data sets were collected from the Servotest TMTS and used as simulation input, especially for the die movement control. The starting condition of the simulation is shown in Figure 5. In the simulation, specific points with defined strain and strain rates are tracked from the beginning to the end to get the thermo-mechanical data of the forging process, which is further used in the multi-class grain size model. This calculates the recrystallization behavior and resulting microstructure in a multi-class system [28] by means of a semi-empirical model.

## 2.3.1. DEFORM Simulation

**Figure 6:**
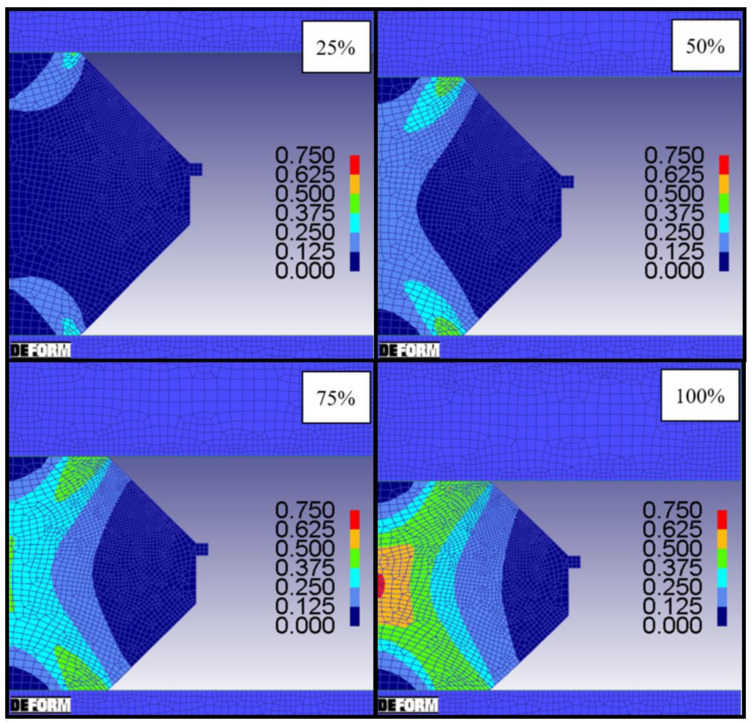
Simulation results at four different stages during the compression test. Local total strain during the double cone experiment: 25%—2.00 mm, 50%—4.00 mm, 75%—6.00 mm and 100%—8.00 mm top die displacement; temperature—1020 °C and strain rate—1 s^−1^.

DEFORM was used as FEM tool to simulate the double cone compression tests, and to determine the local total strains and strain rates. These two-dimensional axisymmetric simulations were carried out as digital shadow from the data of all performed compression tests. A special characteristic of the double cone forging test is the linear evolution of the strain in radial direction with the maximum strain in the center of the specimen and a minimum at the edge. The distribution of the effective strain with increasing die movement is shown in Figure 6. The total deformation of 100% corresponds to a displacement of 8.00 mm. A maximum strain of 0.74 is generated in the center of the specimen. Other evaluated positions are chosen at a radius of 3.31 mm with a strain of 0.54, a radius of 6.55 mm with a strain of 0.27, and a radius of 13.69 mm with a strain of 0.09. Those points have been used in the multi-class grain size model and are analyzed by EBSD measurements to evaluate microstructure and recrystallized fractions.

## 2.3.2. Multi-Class Grain Size Model

**Figure 7:**
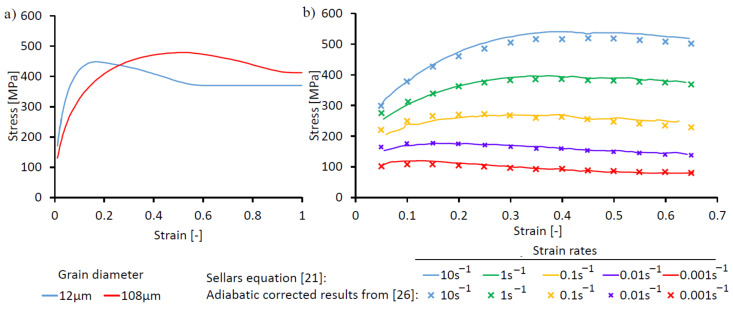
Flow curves at 1000 °C: (**a**) two different grain sizes; (**b**) varying strain rates with Sellars model and experimental data.

The developed multi-class grain size model [28] considers the different strain hardening and softening behaviors of individual classes according to the ASTM grain sizes depending on the mean grain size and strain rates. Because of this adaptation, a series of measured and modelled flow curves (Figure 7) for different grain sizes and strain rates were used to parametrize the semi-empirical model according to the recrystallization conditions and kinetics, as well as initial microstructures. This leads to an earlier recrystallization of smaller grains with lower peak stresses. This can be explained by a faster increase in strain hardening and the fact that the dislocation density relative to the grain volume reaches the recrystallization threshold for DRX earlier.

Further dependencies of the flow curves on strain rate ($\dot{\varphi }$) and temperatures (T) need to be taken into account and are considered in the modeling approach. These grain size-dependent influencing variables are summarized and simulated by means of the temperature compensated strain rate, the Zener–Hollomon parameter (Z[class], Equation (1)). As recrystallization is important during deformation in the low stalking fault material alloy 718, the flow curves are modeled with the Sellars [21] approach and parametrized with measured data from literature [3,19,26]. In Equation (2), the value for the strain is calculated where the peak stress in the flow curve occurs according to the mentioned influencing parameters, wherein the material parameter a_4_ is strain rate dependent. As the material builds necklace bands and duplex microstructures, the value for the strain at peak stress φ_P_ is limited to 0.75, which is relevant for large grain sizes and high strain rates, which led to a satisfying integration of the measured data and literature values.

(1) $$Z\left[\mathrm{class}\right]=\dot{\varphi }\times e^{\frac{Q+(a_{1}\;\times \;\sqrt{D_{0,\mathrm{class}}})}{R\;\times \;T}}$$

(2) $$\varphi_{P}\left[\mathrm{class}\right]={\;a}_{2}\times D_{0,\mathrm{class}}{}^{a_{3}}\times {\dot{\varphi }}^{a_{4}\left(\dot{\varphi }\right)}\times Z{\left[\mathrm{class}\right]}^{a_{5}}$$

In the multi-class version of the semi-empirical model, the actual dislocation density is accumulated in every time increment of the simulation for every grain fraction and compared to a critical stain φ_C_ of the same fraction. By reaching the critical strain φ_C_, which is defined in ratio to the strain at peak stress φ_P_, the formation of recrystallization nuclei for this fraction begins. The amount of recrystallized fraction by overreaching the critical strain and the resulting grain diameter for every class is simulated with Equations (3) and (4). The kinetics (Equation (3)) with experimentally determined strain (φ_0.5_) and strain rates corresponding to the 50% recrystallized volume fraction is represented in this Avrami equation.

(3) $$X_{\mathrm{DRX}}\left[\mathrm{class}\right]=\;1-e^{\ln\left(0.5\right)\;\times \;{\left(\frac{\varphi -\varphi_{C}}{\varphi_{0.5}-\varphi_{C}}\right)}^{a_{6}}}$$

(4) $$D_{\mathrm{DRX}}\left[\mathrm{class}\right]={\;a}_{7}\times {\dot{\varphi }}^{a_{8}}\times Z{\left[\mathrm{class}\right]}^{a_{9}}$$

Based on the resulting diameter and new recrystallized grain fractions, the values get assigned to the corresponding grain class according the ASTM grain sizes as threshold values. If DRX is not completed after deformation, but the critical strain is reached, the simulation enters MDRX, which is modeled with the same approach as DRX. The corresponding Avrami equation for determining the MDRX fractions (Equation (5)) reflects a post-dynamic fraction that depends on a temperature-corrected holding time. The MDRX resulting diameter (Equation (6)) is analog to the DRX.

(5) $$X_{\mathrm{MDRX}}\left[\mathrm{class},\mathrm{time}\right]=\;1-e^{\ln\left(0.5\right)\;\times \;\left(\frac{\sum_{i=1}^{n}t_{i}\;\times {\;e}^{\frac{-Q_{\mathrm{MDRX}}}{R\;\times \;T}}}{b_{1}\;\times {\;\varphi }^{b_{2}}\;\times \;Z{\left[\mathrm{class}\right]}^{b_{3}}}\right)}$$

(6) $$D_{\mathrm{MDRX}}\left[\mathrm{class}\right]={\;b}_{4}\times \varphi^{b_{5}}\times {\dot{\varphi }}^{b_{6}}\times {\left(e^{\frac{-Q_{\mathrm{MDRX}}}{R\;\times \;T}}\right)}^{b_{7}}$$

The material-specific parameters (a and b) from all equation are described more in detail in [3,15]. In multistep production routes, the simulation approach remains, whereas the resulting microstructure describes the initial structure of the following forming step. Special considerations of the remaining strain in terms of the remaining dislocation density in un- or partly recrystallized areas are taken into account.

## 2.4. EBSD Analysis

The double cone experiments performed at temperatures of 980 °C and 1020 °C are analyzed at the 4 points of interest (strains: 0.09, 0.27, 0.54, 0.74) with EBSD with a measured area of 500 × 500 µm and scanned with a step size of 1.00 µm. For preparation, the specimens were embedded in conductive resin and then grinded stepwise with abrasive grinding papers at different grit size levels (up to a grit size of 4000 inch^−2^). Then the specimens were polished with a 3 µm and a 1 µm diamond paste. In order to minimize the effects of previous grinding and polishing processes, the specimens were polished with an alkaline colloidal silica solution (OP-U suspension from Struers) for 20 min and then carefully cleaned with ethanol. The EBSD equipment was a FEI Quanta 250 FEGSEM (Thermo Fisher Scientific Inc., Waltham, MA, USA) equipped with an EDAX AMETEK Hikari XP2 EBSD (AMETEK Materials Analysis Division, Mahwah, NJ, USA) camera, with an acquisition of 15 kV, a spot size of 6 and a 70° tilt angle. The data analysis was performed with the software EDAX-TSL OIM 7.3. (AMETEK Materials Analysis Division, Mahwah, NJ, USA).

**Figure 8:**
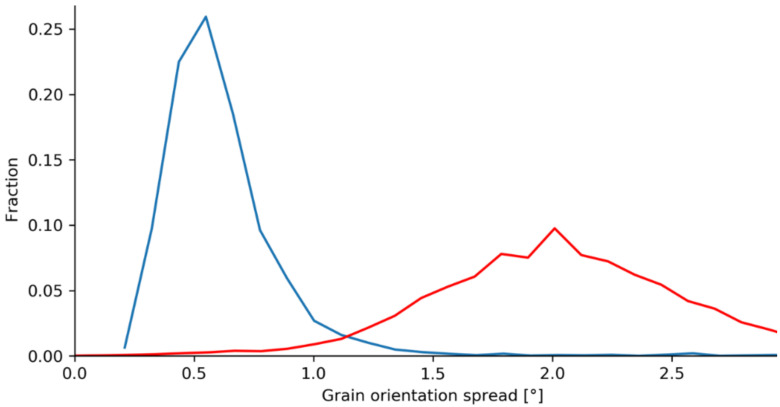
GOS chart-blue: recrystallized microstructure; red: not recrystallized microstructure.

**Figure 9:**
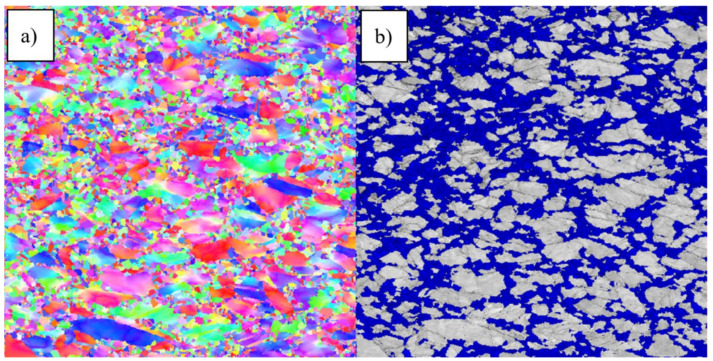
EBSD grain plot (980 °C, 1 s^−1^, strain: 0.54 and 0 s holding time): (**a**) IPF map, (**b**) recrystallized grains colored in blue.

To determine the recrystallized area a Grain Orientation Spread (GOS) angle of 1° was found as most suited to distinguish recrystallized from not recrystallized grains. The determination of this angle is based on a comparison of clearly recrystallized material with strongly deformed and thus not recrystallized material [5,29]. An exemplary correlation between the recrystallized/not recrystallized area fractions and the grain orientation spread is given in Figure 8. Based on the threshold angle all EBSD grain maps were individually analyzed to determine the recrystallized fraction of the measured area. In Figure 9 the recrystallized grains are colored in blue and constitute a fraction of 42.06% of the total area.

## 3. Results

Using EBSD, all Servotest samples forged at 980 °C and 1020 °C with subsequent holding times of 0 s, 5 s and 15 s were evaluated at 4 positions corresponding to strains of interest. The evaluation includes the complete determination of the respective recrystallized portion of the samples and the corresponding ASTM grain size distribution in the 20 selected grain classes from the simulation model.

The samples with 0 s holding time were used to investigate the dynamically recrystallized fractions. In this context, it is necessary to point out that even in this case the recrystallized area also contains a certain meta-dynamic portion, as there is a technically unavoidable delay of ~0.8 s until water quenching starts. This time lag is visible in the results and will be discussed in the following. The MDRX, as well as its kinetics, is determined from the samples with 5 s and 15 s holding time. Besides the recrystallized fractions, the grain size distribution was also evaluated and compared with the simulations results.

### 3.1. Recrystallized Fractions

**Figure 10:**
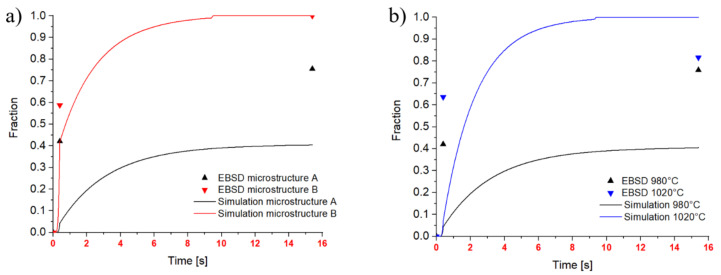
Recrystallized fraction over time including DRX and MDRX: (**a**) Comparison of microstructure A with microstructure B: 980 °C, strain rate 1 s^−1^, strain 0.54; (**b**) Comparison of 980 °C and 1020 °C on microstructure A: strain rate 1 s^−1^, strain 0.54.

The DRX and MDRX fractions are compared separately. The evolution of the recrystallized fraction of the initial microstructures A and B is given in Figure 10. The simulation provides continuous lines, whereas the EBSD data are provided as single results at the end of the forging process and after 15 s of holding time. Figure 10a shows the evolution of the recrystallized fraction during two compression tests with identical test parameters but with the two different initial microstructures. The simulated results for microstructure B corresponding to a fine initial microstructure coincide well with the experimental results. If the delay of the water quenching of 0.8 s is taken into account, the match between the first measurement point and the simulated curve further improves. The delay of the water quenching causes MDRX to begin and the measured recrystallized fraction includes already a certain amount of MDRX. For this reason, the data point could be shifted 0.8 s to the right.

For microstructure A, the simulation clearly shows lower recrystallized fractions than measured. This difference is evaluated in more detail in Figure 10b, which includes data for 980 °C and 1020 °C. This figure illustrates the temperature dependence of MDRX for the coarse initial microstructure A, which is imposed by the model and indicates an unsatisfying model calibration for coarse microstructures. Furthermore, the cut-off to a limit strain is influencing the Avrami approach (Equation (5)) in case of coarse grains.

#### 3.1.1. Dynamic Recrystallized Fractions

**Figure 11:**
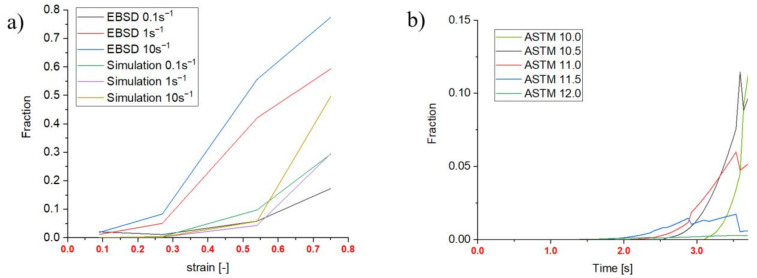
Dynamic recrystallization of microstructure A: (**a**) Recrystallized fractions over strain (980 °C); (**b**) DRX classes 980 °C, 0.1 s^−1^, strain: 0.54.

Regarding DRX fractions, the EBSD data of 0 s holding time after deformation are used. A drift in all results is apparent in Figure 11a due to the delayed quenching after the compression (0.8 s). Therefore, all EBSD measurements provide higher fractions than the simulated ones. However, the tendency of increasing fraction with increasing strain rate is reproduced by the simulation and the experimental analysis at all points of interest (4 different strains: 0.09, 0.27, 0.54 and 0.74). Figure 11b highlights the results and possibilities of the multi-class grain size model, wherein the single ASTM fractions recrystallize into new fractions over time. The different lines mark the resulting ASTM grain size classes during DRX. Recrystallization starts after 1.4 s and until this point only strain hardening and an increase in dislocation density occur. The decrease of some fractions over time marks a transition from one ASTM grain size into another due to the adiabatic heat during the deformation process, leading to a coarser resulting grain diameter.

#### 3.1.2. Meta-Dynamic Recrystallized Fractions

**Figure 12:**
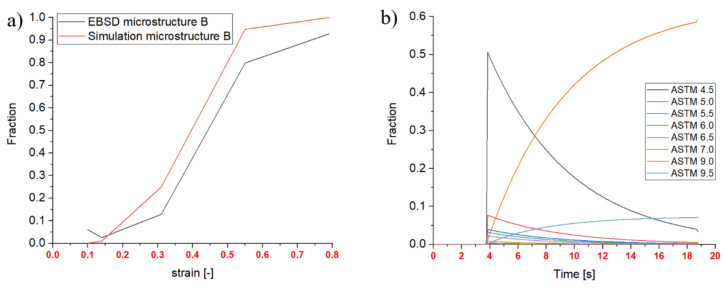
meta-dynamic recrystallization microstructure A: (**a**) Recrystallized fraction over strain, 0.1 s^−1^; (**b**) MDRX classes 980 °C, 0.1 s^−1^, strain: 0.54.

Regarding MDRX, results of 5 s and 15 s holding time are taken into account. Figure 12a shows the recrystallized fractions for the initial microstructure B after 15 s holding time, a global strain rate of 0.1 s^−1^ and 980 °C. An example for a point of interest at a maximum strain of 0.54 is given in Figure 12b, which shows the time-dependent change of the recrystallizing classes in the multi-class grain size model and the disappearing classes due to MDRX. The beginning of the curves at 3.8 s shows the end of deformation and represents the starting values, which are the results from the DRX calculations.

### 3.2. Resulting Grain Size Distribution

**Figure 13:**
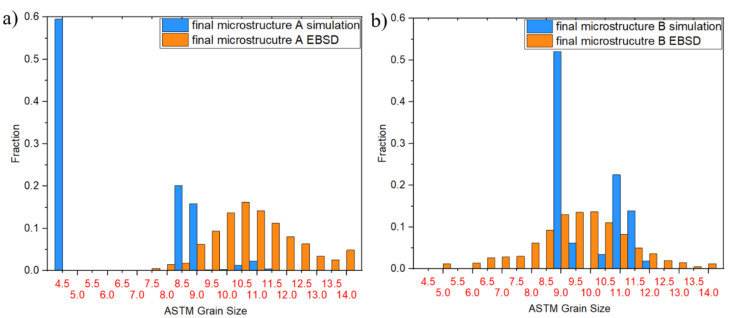
Grain size distribution: strain rate 1 s^−1^, strain 0.54, 15 s holding time; (**a**) microstructure A; (**b**) microstructure B.

As the title “multi-class grain size model” reveals, the resulting microstructures through the DRX and MDRX behavior can be analyzed as distributions. These distributions representing the final microstructures are shown in Figure 13. Evaluating the measured EBSD data, all data sets seem to be normal distributed, whereas the multi-class grain size model shows a peak-like distribution, especially in microstructure B. This is an effect of the double cone experiment with the constant strain rate over time. As the mathematical approach for the resulting grain diameter is strongly dependent on the temperature and strain rate (both nearly constant during the compression), the resulting diameter is also constant and is reflected in the result. The peak of the simulated microstructure B after complete compression and 15 s of holding time is, however, also the mean value of the normal distributed result of the EBSD analysis.

## 4. Discussion

Compared to other models determining a grain size (average single class), the multi-class grain size model provides the possibility to describe real microstructure in an more realistic manner, as for instance bimodal structures can be described. The distribution of the grain sizes, as well as the dependence on grain diameter and strain rates, could be clearly demonstrated by all test series. An important feature of the new model is that it can describe the increase of the number of recrystallization nuclei with increasing strain rates, which causes a shift of the beginning of recrystallization for bigger grains to higher strains.

In case of the initial microstructure B, all experimental and simulated results are in good agreement. For microstructure A, the recrystallization starts too late and the formation of necklace bands around “as large as grains” (ALA grains) found in EBSD images can currently only be considered in the model as phase fractions and not by their actual local distribution in the final microstructure. A further adaptation to an earlier recrystallization start by changing the model parameters would be possible based on targeted experiments. The selection and number of flow curves from literature data as well as the comparison with Gleeble test results and coupling to the flow curve model of Sellars with the introduction of a cutoff limit for the maximum strain at the peak stress proves to be a simple solution for the strongly varying forming parameters.

Another aspect is the differentiation of DRX and MDRX in terms of kinetics and resulting grain diameters. The differences between both phenomena are considered by the multi-class grain size model in terms of the resulting final grain size distribution depending on the forging parameters. In particular, a wide range of forming rates could be simulated with the Servotest, so that the forming equipment used in practice, such as the forging hammer and screw press, can be reproduced. Both machine types are used in the production route of aircraft parts made of alloy 718. Their specific forming characteristics on the recrystallization behavior can be precisely used in the production by means of reaching the demanded final microstructure and thus adjust the associated mechanical properties such as tensile strength and fracture toughness.

Nevertheless, the verification of the DRX at this point is not completely possible with the practical tests, because of the unavoidable delay between the end of the deformation and the beginning of quenching due the technical conditions of the Servotest. Thus, the DRX and MDRX are coupled processes and a minor amount of MDRX fractions is always included in the results. To overcome this problem, an in-situ measurement of the recrystallized fractions and grain sizes during the forming process would be highly beneficial.

The selection of 20 grain classes based on ASTM E112 and the associated threshold values for the individual grain fractions appears to be a good compromise for application, evaluation and standardization. Furthermore, it allows a comparison of the resulting microstructures in accordance with the standard.

In future, the input microstructures should be varied, and thus further distributions should be used as input parameters for verification or adaptation of the multi-class grain size model.

## 5. Conclusions

1. The shift in the DRX is due to delayed water quenching after deformation in all test results. Therefore, the measured recrystallized fraction always includes a certain amount of MDRX. By including the 0.8 s delay in the simulation in case of the tests with 0 s holding time, the fractions are more comparable. Without an in-situ measurement of the microstructure, however, there is no possibility to characterize the condition directly at the end of the forging process.
2. For a coarse initial microstructure, the impact of the temperature on MDRX kinetics is higher than the real double cone results have shown. A further adaptation of model parameters and adapted activation energies based on additional double cone experiments shall be done.
3. The tendencies that smaller grains recrystallize more easily, i.e., at lower strains is reproduced by the multi-class grain size model. In addition, the increasing amount of nucleation spots with higher strain rates is considered in the model with size-dependent threshold values derived from stress maxima of the flow curves.
4. The microstructures of the double cones show normal distributed grain size distributions whereas the multi-class grain size model yields a peak like distribution. This difference can be explained by the advantage of the constant strain rates and temperatures during deformation. As expected, a constant strain rate leads to a homogeneous grain size after recrystallization.

## Figures and Tables

**Table 1:** Chemical composition alloy 718.

Element	Ni [%]	Cr [%]	Fe [%]	Nb [%]	Mo [%]	Ti [%]	Al [%]	Co [%]
Min.	50.00	17.00	-	4.75	2.80	0.65	0.20	-
Max.	55.00	21.00	Balance	5.50	3.30	1.15	0.80	1.00

**Table 2:** Testing parameters for double cone compression tests.

Parameter	Values
Temperatures (isothermal)	900 °C, 980 °C, 1000 °C, 1020 °C, 1050 °C
Strain rates	0.1 s^−1^, 1.0 s^−1^, 10.0 s^−1^, 50 s^−1^
Holding times	0 s, 5 s, 15 s
Initial microstructures	2 different distributions: Microstructure A (coarse) and B (fine)

## Data Availability

The data presented in this study are available on request from the corresponding author. The data are not publicly available due to restrictions from industrial partners.
